# Extended Divergence on a Foliation by Continuous-Type Escort Distributions

**DOI:** 10.3390/e28060629

**Published:** 2026-06-02

**Authors:** Keiko Uohashi

**Affiliations:** Faculty of Engineering, Tohoku Gakuin University, Sendai 984-8588, Japan; uohashi@mail.tohoku-gakuin.ac.jp

**Keywords:** escort distribution, exponential family, foliation, dualistic structure, divergence, relative entropy, nonextensive statistics, information geometry, affine geometry

## Abstract

From an information geometric perspective, this study considers a natural foliation of dualistic structures associated with escort distributions of exponential families. We propose an extended divergence on this foliation by continuous-type escort distributions. Specifically, we investigate the foliation formed by escort distributions to analyze the transition of *q*-parameters, rather than relying on a fixed parameter. Within this foliation, distinct *q*-parameters and their corresponding dualistic α-parameters were defined on each leaf. Finally, we present a decomposition of the extended divergence on this foliation, providing an analog to the method previously established for discrete escort distributions.

## 1. Introduction

The Cauchy and Student’s *t*-distributions are prototypical examples of *q*-Gaussian distributions. Thus, the set of *q*-normal distributions is regarded as a standard *q*-exponential family, underlining its strong connection to nonextensive statistical mechanics [1,2]. Both *q*-normal distributions and *q*-exponential families have been extensively studied from an information-geometric perspective, with applications ranging from nonextensive statistical mechanics to various other fields [3,4,5,6,7,8,9,10]. Furthermore, deformed *q*-exponential families, defined by deformed logarithm and exponential functions, have been applied to escort distributions [11], and their Hessian and conformal structures have been actively investigated [12,13,14].

Our previous work explored the foliation formed by deformed probability simplexes representing sets of escort distributions (a typical discrete case of *q*-exponential families) in relation to the continuous variation of α-parameters within the information geometry framework [15,16]. For example, we define an extended divergence and its decomposition on this foliation. We then extend this analysis to the foliation formed by escort distributions associated with continuous probability distributions [17]. In this study, we augment the mathematical proofs provided in our previous work and elucidate the foliation structure formed by escort distributions associated with normal distributions.

The paper is organized as follows. First, we introduce an α-divergence on a subset of an affine space identified with a foliation of multiplied exponential families and discuss the relationship with the Tsallis relative entropy. Next, we present the component expressions of the Riemannian metric, α-affine connections, and α-coordinates introduced from the α-divergence. We compare α-divergences for continuous and discrete probability distributions. We also describe the dualistic structures of affine immersions as level surfaces on the *q*-escort distributions of exponential families. Then, we define the extended divergence on a foliation formed by *q*-escort distributions of an exponential distribution. Finally, we propose the decomposition of the resulting extended divergence on the foliation.

## 2. α-Divergences on a Foliation of Multiplied Exponential Families

Following the characterization of discrete probability distributions in [12,13], we explain the α-divergences on a subset of an affine space by the foliation of probability distributions.

Let $S_{1}$ be an exponential family defined by

(1) $$S_{1}=\left\{p\left(x;\theta_{\left(1\right)}\right)\;|\;p\left(x;\theta_{\left(1\right)}\right)=\exp\left(\sum_{i=1}^{n}\theta_{\left(1\right)}^{i}c_{i}\left(x\right)-\Phi \left(\theta_{\left(1\right)}\right)\right),\;\theta_{\left(1\right)}\in \Theta_{\left(1\right)}\subset R^{n}\right\},$$

where $\theta_{\left(1\right)}=\left(\theta_{\left(1\right)}^{1},\cdots ,\theta_{\left(1\right)}^{n}\right)$ is an element of the parameter space $\Theta_{\left(1\right)}$, Φ is a function on $\Theta_{\left(1\right)}$, and $c_{1},\cdots ,c_{n}$ are functions on the sample space X⊂R. Let $\Theta_{\left(1\right)}$ be a smooth submanifold of $R^{n}$, identified with a smooth submanifold of an *n*-dimensional affine space $A^{n}$. Let $\Theta_{\left(u\right)}$ be the parameter space, which is the *u* times of $\Theta_{\left(1\right)}$, i.e.,

(2) $$\Theta_{\left(u\right)}=\left\{\theta_{\left(u\right)}=\left(\theta_{\left(u\right)}^{1},\cdots ,\theta_{\left(u\right)}^{n}\right)\;|\;\theta_{\left(u\right)}=u\theta_{\left(1\right)}=\left(u\theta_{\left(1\right)}^{1},\cdots ,u\theta_{\left(1\right)}^{n}\right),\;\theta_{\left(1\right)}\in \Theta_{\left(1\right)}\right\},\;\;u\in A_{+}.$$

**Figure 1:**
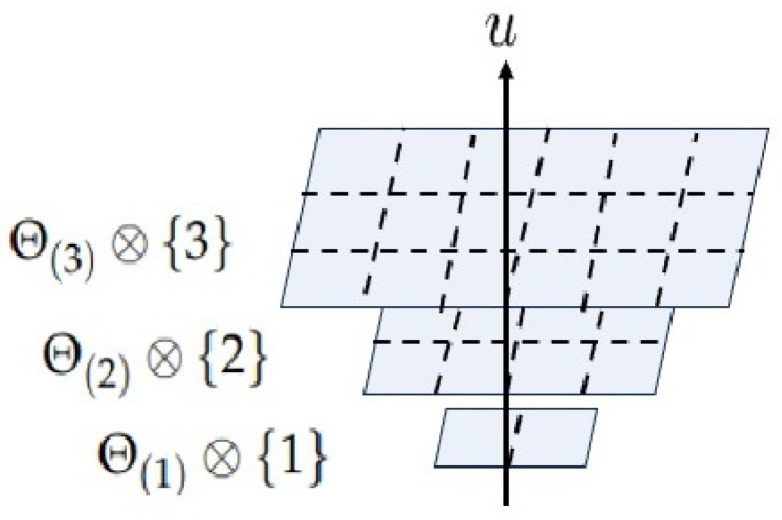
An extended parameter space Θ (displaying cases for u=1,2, and 3).

We consider an extended parameter space $\Theta \equiv {⋃}_{u>0}\;\left(\Theta_{\left(u\right)}⊗\left\{u\right\}\right)$, which is naturally identified by a smooth submanifold of $A^{n}⊗A_{+}$ (Figure 1). Defining the cone-like Θ using the parameter u is for the purpose of defining projections and immersions of an exponential family in later sections. In the following, all integrations are performed over the sample space X.

**Definition 1** 
([17])**.** *Let $\Theta_{\left(1\right)}$ be the parameter space of an exponential family, and $\Theta_{\left(u\right)}$ the u times of $\Theta_{\left(1\right)}$. For −1<α<1, an α-divergence $D^{\left(\alpha \right)}$ on an extended parameter space $\Theta :={⋃}_{u>0}\;\left(\Theta_{\left(u\right)}⊗\left\{u\right\}\right)\subset A^{n}⊗A_{+}$ is defined by*

(3) $$D^{\left(\alpha \right)}\left(a,b\right)=\frac{4}{1-\alpha^{2}}\left\{\frac{1-\alpha }{2}u_{a}+\frac{1+\alpha }{2}u_{b}-\int {\left(u_{a}p\left(x;\frac{1}{u_{a}}\theta_{a}\right)\right)}^{\frac{1-\alpha }{2}}{\left(u_{b}p\left(x;\frac{1}{u_{b}}\theta_{b}\right)\right)}^{\frac{1+\alpha }{2}}dx\right\},$$

*where $a=\left(\theta_{a},u_{a}\right),b=\left(\theta_{b},u_{b}\right)\in \Theta$. $\theta_{a}\in \Theta_{\left(u_{a}\right)},\theta_{b}\in \Theta_{\left(u_{b}\right)}$, and $u_{a},u_{b}\in A_{+}$.*

However, an α-divergence $D^{\left(\alpha \right)}$ is defined when the integral in Equation (3) exists. From this definition, the following properties hold.

**Proposition 1** 
([17])**.** *An α-divergence $D^{\left(\alpha \right)}$ on an extended parameter space* Θ *satisfies the following:*

*(i)*  *In the case of $\theta_{a},\theta_{b}\in \Theta_{\left(u_{a}\right)}$, i.e., $u_{a}=u_{b}\in A_{+}$,*
  (4) $$D^{\left(\alpha \right)}\left(a,b\right)=u_{a}D^{\left(\alpha \right)}\left(\left(\frac{1}{u_{a}}\theta_{a},1\right),\left(\frac{1}{u_{a}}\theta_{b},1\right)\right).$$
*(ii)*  *In the case of $\left(1/u_{b}\right)\theta_{b}=\left(1/u_{c}\right)\theta_{c}\in \Theta_{\left(1\right)}$,*
  (5) $$D^{\left(\alpha \right)}\left(b,c\right)=\frac{4}{1-\alpha^{2}}\left(\frac{1-\alpha }{2}u_{b}+\frac{1+\alpha }{2}u_{c}-u_{b}^{\frac{1-\alpha }{2}}\;u_{c}^{\frac{1+\alpha }{2}}\right).$$

The proof of Proposition 1 follows directly from the normalization condition:

$$\int p\left(x;\frac{1}{u_{a}}\theta_{a}\right)dx=\int p\left(x;\frac{1}{u_{b}}\theta_{b}\right)dx=1,\;\;\;\;\;\;\frac{1}{u_{a}}\theta_{a},\;\;\frac{1}{u_{b}}\theta_{b}\in \Theta_{\left(1\right)}.$$

Regarding the decomposition of the α-divergence $D^{\left(\alpha \right)}$, we obtain the following theorem.

**Theorem 1.** 
*For −1<α<1, an α-divergence $D^{\left(\alpha \right)}$ on an extended parameter space $\Theta :={⋃}_{u>0}\;\left(\Theta_{\left(u\right)}⊗\left\{u\right\}\right)\subset A^{n}⊗A_{+}$ satisfies that*

(6) $$D^{\left(\alpha \right)}\left(a,c\right)=\mu \;D^{\left(\alpha \right)}\left(a,b\right)+D^{\left(\alpha \right)}\left(b,c\right),\;\;\;\;\mu ={\left(\frac{u_{c}}{u_{b}}\right)}^{\frac{1+\alpha }{2}},$$

*if $a=\left(\theta_{a},u_{a}\right),\;b=\left(\theta_{b},u_{b}\right),\;c=\left(\theta_{c},u_{c}\right)\in \Theta$, $u_{a}=u_{b}$, and $\left(1/u_{b}\right)\theta_{b}=\left(1/u_{c}\right)\theta_{c}$.*

**Proof.**  Each term is defined as follows:

$$\begin{array}{ccc} \frac{1-\alpha^{2}}{4}D^{\left(\alpha \right)}\left(a,c\right) & = & \frac{1-\alpha }{2}u_{a}+\frac{1+\alpha }{2}u_{c}-\int {\left(u_{a}p\left(x;\frac{1}{u_{a}}\theta_{a}\right)\right)}^{\frac{1-\alpha }{2}}{\left(u_{c}p\left(x;\frac{1}{u_{c}}\theta_{c}\right)\right)}^{\frac{1+\alpha }{2}}dx\\ & = & \frac{1-\alpha }{2}u_{b}+\frac{1+\alpha }{2}u_{c}-u_{b}^{\frac{1-\alpha }{2}}\cdot \mu \;u_{b}^{\frac{1+\alpha }{2}}\int p{\left(x;\frac{1}{u_{a}}\theta_{a}\right)}^{\frac{1-\alpha }{2}}p{\left(x;\frac{1}{u_{c}}\theta_{c}\right)}^{\frac{1+\alpha }{2}}dx\\ & = & \frac{1-\alpha }{2}u_{b}+\frac{1+\alpha }{2}u_{c}-\mu \;u_{b}\int p{\left(x;\frac{1}{u_{a}}\theta_{a}\right)}^{\frac{1-\alpha }{2}}p{\left(x;\frac{1}{u_{b}}\theta_{b}\right)}^{\frac{1+\alpha }{2}}dx. \end{array}$$

$$\begin{array}{ccc} \frac{1-\alpha^{2}}{4}D^{\left(\alpha \right)}\left(a,b\right) & = & \frac{1-\alpha }{2}u_{a}+\frac{1+\alpha }{2}u_{b}-\int {\left(u_{a}p\left(x;\frac{1}{u_{a}}\theta_{a}\right)\right)}^{\frac{1-\alpha }{2}}{\left(u_{b}p\left(x;\frac{1}{u_{b}}\theta_{b}\right)\right)}^{\frac{1+\alpha }{2}}dx\\ & = & \frac{1-\alpha }{2}u_{b}+\frac{1+\alpha }{2}u_{b}-u_{b}^{\frac{1-\alpha }{2}}u_{b}^{\frac{1+\alpha }{2}}\int p{\left(x;\frac{1}{u_{a}}\theta_{a}\right)}^{\frac{1-\alpha }{2}}p{\left(x;\frac{1}{u_{b}}\theta_{b}\right)}^{\frac{1+\alpha }{2}}dx\\ & = & u_{b}-u_{b}\int p{\left(x;\frac{1}{u_{a}}\theta_{a}\right)}^{\frac{1-\alpha }{2}}p{\left(x;\frac{1}{u_{b}}\theta_{b}\right)}^{\frac{1+\alpha }{2}}dx. \end{array}$$

$$\begin{array}{ccc} \frac{1-\alpha^{2}}{4}D^{\left(\alpha \right)}\left(b,c\right) & = & \frac{1-\alpha }{2}u_{b}+\frac{1+\alpha }{2}u_{c}-\int {\left(u_{b}p\left(x;\frac{1}{u_{b}}\theta_{b}\right)\right)}^{\frac{1-\alpha }{2}}{\left(u_{c}p\left(x;\frac{1}{u_{c}}\theta_{c}\right)\right)}^{\frac{1+\alpha }{2}}dx\\ & = & \frac{1-\alpha }{2}u_{b}+\frac{1+\alpha }{2}u_{c}-\mu \;u_{b}\int p\left(x;\frac{1}{u_{b}}\theta_{b}\right)dx\\ & = & \frac{1-\alpha }{2}u_{b}+\frac{1+\alpha }{2}u_{c}-\mu \;u_{b}. \end{array}$$

Then, we have that

$$\frac{1-\alpha^{2}}{4}\left\{D^{\left(\alpha \right)}\left(a,c\right)-\left(\mu D^{\left(\alpha \right)}\left(a,b\right)+D^{\left(\alpha \right)}\left(b,c\right)\right)\right\}=-\mu \;u_{b}+\mu \;u_{b}=0.$$

Thus, Theorem 1 holds. □

By Theorem 1, the next proposition holds.

**Proposition 2.** 
*For −1<α<1, let $D^{\left(\alpha \right)}$ be an α-divergence on an extended parameter space $\Theta :={⋃}_{u>0}\;\left(\Theta_{\left(u\right)}⊗\left\{u\right\}\right)\subset A^{n}⊗A_{+}$. For a,b∈Θ, it holds that*

(7) $$D^{\left(\alpha \right)}\left(a,b\right)\geq 0.$$

*Furthermore, $D^{\left(\alpha \right)}\left(a,b\right)=0$ if and only if a=b,*

**Proof.**  Let $a=\left(\theta_{a},u_{a}\right),\;b=\left(\theta_{b},u_{b}\right)\in \Theta$ and $\mu ={\left(u_{b}/u_{a}\right)}^{\frac{1+\alpha }{2}}$. For $\tilde{b}=\left(\left(u_{a}/u_{b}\right)\;\theta_{b},u_{a}\right)\in \Theta$, it follows from Theorem 1 that

(8) $$D^{\left(\alpha \right)}\left(a,b\right)=\mu \;D^{\left(\alpha \right)}\left(a,\tilde{b}\right)+D^{\left(\alpha \right)}\left(\tilde{b},b\right).$$

From Proposition 1 (i), we have

(9) $$D^{\left(\alpha \right)}\left(a,\tilde{b}\right)=u_{a}\;D^{\left(\alpha \right)}\left(\left(\frac{1}{u_{a}}\theta_{a},1\right),\left(\frac{1}{u_{a}}\theta_{\tilde{b}},1\right)\right).$$

The α-divergence $D^{\left(\alpha \right)}$ on the right-hand side of Equation (9) is the divergence on the exponential family $S_{1}$ and satisfies

(10) $$D^{\left(\alpha \right)}\left(\left(\frac{1}{u_{a}}\theta_{a},1\right),\left(\frac{1}{u_{a}}\theta_{\tilde{b}},1\right)\right)=\frac{4}{1-\alpha^{2}}\left\{1-\int p{\left(x;\frac{1}{u_{a}}\theta_{a}\right)}^{\frac{1-\alpha }{2}}p{\left(x;\frac{1}{u_{a}}\theta_{\tilde{b}}\right)}^{\frac{1+\alpha }{2}}dx\right\}.$$

Therefore, $D^{\left(\alpha \right)}\left(a,\tilde{b}\right)\geq 0$, where equality holds if and only if $a=\tilde{b}$. Similarly, from Proposition 1 (ii), it holds that

(11) $$D^{\left(\alpha \right)}\left(\tilde{b},b\right)=\frac{4}{1-\alpha^{2}}\left(\frac{1-\alpha }{2}u_{a}+\frac{1+\alpha }{2}u_{b}-u_{a}^{\frac{1-\alpha }{2}}u_{b}^{\frac{1+\alpha }{2}}\right).$$

The right-hand side of Equation (11) is an α-divergence on the half-line {u∣u>0}. Thus, $D^{\left(\alpha \right)}\left(\tilde{b},b\right)\geq 0$, where equality holds if and only if $\tilde{b}=b$. Consequently, Proposition 2 holds. □

At the end of this section, we describe the relationship between the α-divergence and entropy parameterized by *q*. For q=(1−α)/2, the divergence $D^{\left(\alpha \right)}$ restricted to the exponential family $S_{1}$ satisfies

(12) $$D^{\left(\alpha \right)}\left(a,b\right)=\frac{1}{q}K_{q}\left(p\left(x;\theta_{a}\right),p\left(x;\theta_{b}\right)\right),\;a=\left(\theta_{a},1\right),\;b=\left(\theta_{b},1\right)\in \Theta_{\left(1\right)}⊗\left\{1\right\},$$

where $K_{q}$ is the continuous Tsallis relative entropy defined by

$$\begin{array}{ccc} K_{q}\left(p\left(x;\theta_{a}\right),p\left(x;\theta_{b}\right)\right) & \equiv & -\int p\left(x;\theta_{a}\right)\ln_{q}\left(\frac{p(x;\theta_{b})}{p(x;\theta_{a})}\right)dx\\ & = & \frac{1}{1-q}\left\{1-\int p{\left(x;\theta_{a}\right)}^{q}p{\left(x;\theta_{b}\right)}^{1-q}dx\right\},\\ & & p\left(x;\theta_{a}\right),\;p\left(x;\theta_{b}\right)\in S_{1}, \end{array}$$

with $p\left(x;\theta_{a}\right),p\left(x;\theta_{b}\right)\in S_{1}$, and $\ln_{q}$ is the *q*-logarithm defined by

(13) $$\ln_{q}x\equiv \frac{x^{1-q}-1}{1-q},\;q\neq 1,\;x>0.$$

The Tsallis relative entropy $K_{q}$ converges to the Kullback–Leibler divergence as q→1 because $\lim_{q\to 1}\ln_{q}x=\log x$. Similarly, the α-divergence $D^{\left(\alpha \right)}$ converges to the Kullback–Leibler divergence as α→−1 on the exponential family $S_{1}$ [1,2,12,13].

## 3. The Dualistic Structure Induced by an α-Divergence on an Extended Parameter Space

The α-divergence on the extended parameter space $\Theta :={⋃}_{u>0}\left(\Theta_{\left(u\right)}⊗\left\{u\right\}\right)$ induces a dualistic structure.

Let $\left\{\theta^{1},\cdots ,\theta^{n},u\right\}$ be the canonical affine coordinate system on $\Theta \subset A^{n}⊗A_{+}$ such that its restriction to $\Theta_{\left(u\right)}$ coincides with $\left\{\theta_{\left(u\right)}^{1},\cdots ,\theta_{\left(u\right)}^{n},u\right\}$. We define a vector field transversal to each $\Theta_{\left(u\right)}⊗\left\{u\right\}$ by

(14) $${\left.\frac{\partial }{\partial \bar{u}}\right|}_{a}=\frac{1}{u_{a}}\sum_{i=1}^{n}\theta_{a}^{i}\frac{\partial }{\partial \theta^{i}}+\frac{\partial }{\partial u}.$$

**Figure 2:**
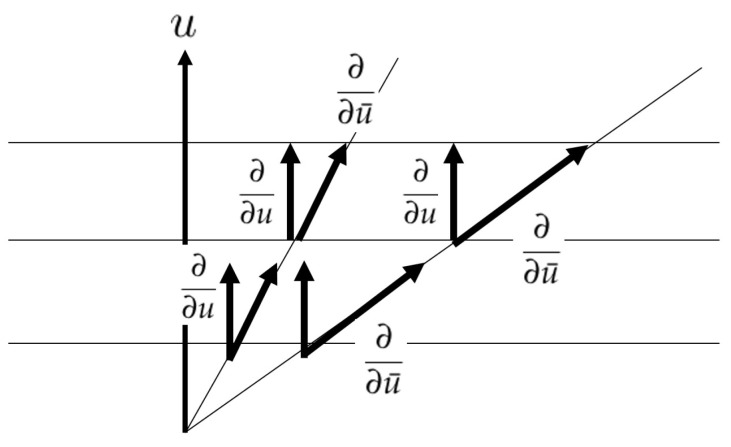
Transversal vector fields.

Note that $\left(\partial /\partial \bar{u}\right)≢\left(\partial /\partial u\right)$ by Equation (14). However, the coordinate $\bar{u}$ is determined such that $\bar{u}\left(a\right)=u\left(a\right)$ for all a∈Θ (Figure 2).

Then, the Riemannian metric *g* and α-connection $\nabla^{\left(\alpha \right)}$ are computed as follows:

$$\begin{array}{ccc} & & g{\left(\frac{\partial }{\partial \theta^{i}},\frac{\partial }{\partial \theta^{j}}\right)}_{a}=-{\left(\frac{\partial }{\partial \theta^{i}}\right)}_{a}{\left(\frac{\partial }{\partial \theta^{j}}\right)}_{b}D^{\left(\alpha \right)}\left(a,b\right){|}_{a=b}\\ & = & \frac{1}{u_{a}}\int p\left(x;\frac{1}{u_{a}}\theta_{a}\right)\left(c_{i}\left(x\right)-\frac{\partial \Phi (\theta_{\left(1\right)})}{\partial \theta_{\left(1\right)}^{i}}{|}_{\theta_{\left(1\right)}=\frac{1}{u_{a}}\theta_{a}}\right)\left(c_{j}\left(x\right)-\frac{\partial \Phi (\theta_{\left(1\right)})}{\partial \theta_{\left(1\right)}^{j}}{|}_{\theta_{\left(1\right)}=\frac{1}{u_{a}}\theta_{a}}\right)\;dx, \end{array}$$

$$\begin{array}{ccc} g{\left(\frac{\partial }{\partial \theta^{i}},\frac{\partial }{\partial \bar{u}}\right)}_{a} & = & g{\left(\frac{\partial }{\partial \bar{u}},\frac{\partial }{\partial \theta^{i}}\right)}_{a}=-{\left(\frac{\partial }{\partial \theta^{i}}\right)}_{a}{\left(\frac{\partial }{\partial \bar{u}}\right)}_{b}D^{\left(\alpha \right)}\left(a,b\right){|}_{a=b}\\ & = & \frac{1}{u_{a}}\int p\left(x;\frac{1}{u_{a}}\theta_{a}\right)\left(c_{i}\left(x\right)-\frac{\partial \Phi (\theta_{\left(1\right)})}{\partial \theta_{\left(1\right)}^{i}}{|}_{\theta_{\left(1\right)}=\frac{1}{u_{a}}\theta_{a}}\right)\;dx, \end{array}$$

$$g{\left(\frac{\partial }{\partial \bar{u}},\frac{\partial }{\partial \bar{u}}\right)}_{a}=-{\left(\frac{\partial }{\partial \bar{u}}\right)}_{a}{\left(\frac{\partial }{\partial \bar{u}}\right)}_{b}D^{\left(\alpha \right)}\left(a,b\right){|}_{a=b}=\frac{1}{u_{a}},$$

$$\begin{array}{ccc} & & g{\left(\nabla_{\frac{\partial }{\partial \theta^{i}}}^{\left(\alpha \right)}\frac{\partial }{\partial \theta^{j}},\frac{\partial }{\partial \theta^{k}}\right)}_{a}=-{\left(\frac{\partial^{2}}{\partial \theta^{i}\partial \theta^{j}}\right)}_{a}{\left(\frac{\partial }{\partial \theta^{k}}\right)}_{b}D^{\left(\alpha \right)}\left(a,b\right){|}_{a=b}\\ & = & \frac{1}{u_{a}^{2}}\int p\left(x;\frac{1}{u_{a}}\theta_{a}\right)\{\frac{1-\alpha }{2}\left(c_{i}\left(x\right)-\frac{\partial \Phi (\theta_{\left(1\right)})}{\partial \theta_{\left(1\right)}^{i}}{|}_{\theta_{\left(1\right)}=\frac{1}{u_{a}}\theta_{a}}\right)\left(c_{j}\left(x\right)-\frac{\partial \Phi (\theta_{\left(1\right)})}{\partial \theta_{\left(1\right)}^{j}}{|}_{\theta_{\left(1\right)}=\frac{1}{u_{a}}\theta_{a}}\right)\\ & & -\frac{\partial^{2}\Phi \left(\theta_{\left(1\right)}\right)}{\partial \theta_{\left(1\right)}^{i}\partial \theta_{\left(1\right)}^{j}}{|}_{\theta_{\left(1\right)}=\frac{1}{u_{a}}\theta_{a}}\}\left(c_{k}\left(x\right)-\frac{\partial \Phi (\theta_{\left(1\right)})}{\partial \theta_{\left(1\right)}^{k}}{|}_{\theta_{\left(1\right)}=\frac{1}{u_{a}}\theta_{a}}\right)\;dx, \end{array}$$

$$\begin{array}{ccc} & & g{\left(\nabla_{\frac{\partial }{\partial \theta^{i}}}^{\left(\alpha \right)}\frac{\partial }{\partial \theta^{j}},\frac{\partial }{\partial \bar{u}}\right)}_{a}=-{\left(\frac{\partial^{2}}{\partial \theta^{i}\partial \theta^{j}}\right)}_{a}{\left(\frac{\partial }{\partial \bar{u}}\right)}_{b}D^{\left(\alpha \right)}\left(a,b\right){|}_{a=b}\\ & = & \frac{1}{u_{a}^{2}}\int p\left(x;\frac{1}{u_{a}}\theta_{a}\right)\{\frac{1-\alpha }{2}\left(c_{i}\left(x\right)-\frac{\partial \Phi (\theta_{\left(1\right)})}{\partial \theta_{\left(1\right)}^{i}}{|}_{\theta_{\left(1\right)}=\frac{1}{u_{a}}\theta_{a}}\right)\left(c_{j}\left(x\right)-\frac{\partial \Phi (\theta_{\left(1\right)})}{\partial \theta_{\left(1\right)}^{j}}{|}_{\theta_{\left(1\right)}=\frac{1}{u_{a}}\theta_{a}}\right)\\ & & -\frac{\partial^{2}\Phi \left(\theta_{\left(1\right)}\right)}{\partial \theta_{\left(1\right)}^{i}\partial \theta_{\left(1\right)}^{j}}{|}_{\theta_{\left(1\right)}=\frac{1}{u_{a}}\theta_{a}}\}\;dx, \end{array}$$

$$\begin{array}{ccc} g{\left(\nabla_{\frac{\partial }{\partial \bar{u}}}^{\left(\alpha \right)}\frac{\partial }{\partial \theta^{i}},\frac{\partial }{\partial \theta^{j}}\right)}_{a} & = & g{\left(\nabla_{\frac{\partial }{\partial \theta^{i}}}^{\left(\alpha \right)}\frac{\partial }{\partial \bar{u}},\frac{\partial }{\partial \theta^{j}}\right)}_{a}=-{\left(\frac{\partial^{2}}{\partial \bar{u}\partial \theta^{i}}\right)}_{a}{\left(\frac{\partial }{\partial \theta^{j}}\right)}_{b}D^{\left(\alpha \right)}\left(a,b\right){|}_{a=b}\\ & = & -\frac{1+\alpha }{2}\frac{1}{u_{a}^{2}}\int p\left(x;\frac{1}{u_{a}}\theta_{a}\right)\left(c_{i}\left(x\right)-\frac{\partial \Phi (\theta_{\left(1\right)})}{\partial \theta_{\left(1\right)}^{i}}{|}_{\theta_{\left(1\right)}=\frac{1}{u_{a}}\theta_{a}}\right)\\ & & \left(c_{j}\left(x\right)-\frac{\partial \Phi (\theta_{\left(1\right)})}{\partial \theta_{\left(1\right)}^{j}}{|}_{\theta_{\left(1\right)}=\frac{1}{u_{a}}\theta_{a}}\right)\;dx, \end{array}$$

$$\begin{array}{ccc} g{\left(\nabla_{\frac{\partial }{\partial \bar{u}}}^{\left(\alpha \right)}\frac{\partial }{\partial \bar{u}},\frac{\partial }{\partial \theta^{i}}\right)}_{a} & = & -{\left(\frac{\partial^{2}}{\partial {\bar{u}}^{2}}\right)}_{a}{\left(\frac{\partial }{\partial \theta^{i}}\right)}_{b}D^{\left(\alpha \right)}\left(a,b\right){|}_{a=b}\\ & = & -\frac{1+\alpha }{2}\frac{1}{u_{a}^{2}}\int p\left(x;\frac{1}{u_{a}}\theta_{a}\right)\left(c_{i}\left(x\right)-\frac{\partial \Phi (\theta_{\left(1\right)})}{\partial \theta_{\left(1\right)}^{i}}{|}_{\theta_{\left(1\right)}=\frac{1}{u_{a}}\theta_{a}}\right)dx, \end{array}$$

$$\begin{array}{ccc} g{\left(\nabla_{\frac{\partial }{\partial \bar{u}}}^{\left(\alpha \right)}\frac{\partial }{\partial \theta^{i}},\frac{\partial }{\partial \bar{u}}\right)}_{a} & = & g{\left(\nabla_{\frac{\partial }{\partial \theta^{i}}}^{\left(\alpha \right)}\frac{\partial }{\partial \bar{u}},\frac{\partial }{\partial \bar{u}}\right)}_{a}=-{\left(\frac{\partial^{2}}{\partial \bar{u}\partial \theta^{i}}\right)}_{a}{\left(\frac{\partial }{\partial \bar{u}}\right)}_{b}D^{\left(\alpha \right)}\left(a,b\right){|}_{a=b}\\ & = & -\frac{1+\alpha }{2}\frac{1}{u_{a}^{2}}\int p\left(x;\frac{1}{u_{a}}\theta_{a}\right)\left(c_{i}\left(x\right)-\frac{\partial \Phi (\theta_{\left(1\right)})}{\partial \theta_{\left(1\right)}^{i}}{|}_{\theta_{\left(1\right)}=\frac{1}{u_{a}}\theta_{a}}\right)\;dx, \end{array}$$

and

$$g{\left(\nabla_{\frac{\partial }{\partial \bar{u}}}^{\left(\alpha \right)}\frac{\partial }{\partial \bar{u}},\frac{\partial }{\partial \bar{u}}\right)}_{a}=-{\left(\frac{\partial^{2}}{\partial {\bar{u}}^{2}}\right)}_{a}{\left(\frac{\partial }{\partial \bar{u}}\right)}_{b}D^{\left(\alpha \right)}\left(a,b\right){|}_{a=b}=-\frac{1+\alpha }{2}\frac{1}{u_{a}^{2}},$$

where $D^{\left(\alpha \right)}$ is the α-divergence defined by Equation (3), and a,b∈Θ. Since *p* belongs to an exponential family, the following equation is used for the calculations above.

$$\begin{array}{ccc} \frac{\partial }{\partial \theta^{i}}p\left(x;\frac{1}{u}\theta \right){|}_{a} & = & p\left(x;\frac{1}{u_{a}}\theta_{a}\right)\frac{\partial }{\partial \theta_{\left(1\right)}^{i}}\left(\sum_{i=1}^{n}\theta_{\left(1\right)}^{i}c_{i}\left(x\right)-\Phi \left(\theta_{\left(1\right)}\right)\right){|}_{\theta_{\left(1\right)}=\frac{1}{u_{a}}\theta_{a}}\;\frac{\partial }{\partial \theta^{i}}\left(\frac{1}{u}\theta^{i}\right){|}_{a}\\ & = & \frac{1}{u_{a}}p\left(x;\frac{1}{u_{a}}\theta_{a}\right)\left(c_{i}\left(x\right)-\frac{\partial \Phi (\theta_{\left(1\right)})}{\partial \theta_{\left(1\right)}^{i}}{|}_{\theta_{\left(1\right)}=\frac{1}{u_{a}}\theta_{a}}\right) \end{array}$$

The triplet $\left(\Theta ,\nabla^{\left(\alpha \right)},g\right)$ forms a statistical manifold, and $\left(\Theta ,\nabla^{\left(-\alpha \right)},g\right)$ is its dual.

The α-coordinate system $\left\{\theta^{(\alpha )1},\ldots ,\theta^{(\alpha )n},{\bar{u}}^{\left(\alpha \right)}\right\}$ on Θ is given as follows:

$$\theta^{(\alpha )i}\left(a\right)=\int \frac{\partial }{\partial \theta^{i}}\ln_{1-q}\left\{up\left(x;\frac{1}{u}\theta \right)\right\}\;dx{|}_{a}=\frac{2}{1-\alpha }\frac{\partial }{\partial \theta^{i}}\int \left\{{\left(up\left(x;\frac{1}{u}\theta \right)\right)}^{\frac{1-\alpha }{2}}-1\right\}\;dx{|}_{a}$$

(15) $$=\frac{1}{u_{a}}\int {\left(u_{a}p\left(x;\frac{1}{u_{a}}\theta_{a}\right)\right)}^{\frac{1-\alpha }{2}}\left(c_{i}\left(x\right)-\frac{\partial \Phi (\theta_{\left(1\right)})}{\partial \theta_{\left(1\right)}^{i}}{|}_{\theta_{\left(1\right)}=\frac{1}{u_{a}}\theta_{a}}\right)\;dx,\;\;\;\;i=1,\cdots ,n,$$

$${\bar{u}}^{\left(\alpha \right)}\left(a\right)=\int \frac{\partial }{\partial \bar{u}}\ln_{1-q}\left\{up\left(x;\frac{1}{u}\theta \right)\right\}\;dx{|}_{a}=\frac{2}{1-\alpha }\int \frac{\partial }{\partial \bar{u}}\left\{{\left(up\left(x;\frac{1}{u}\theta \right)\right)}^{\frac{1-\alpha }{2}}-1\right\}\;dx{|}_{a}$$

(16) $$=\frac{1}{u_{a}}\int {\left(u_{a}p\left(x;\frac{1}{u_{a}}\theta_{a}\right)\right)}^{\frac{1-\alpha }{2}}\;dx,\;\;\;\;\;\;\;\;a\in \Theta ,\;\;\;\;q=\frac{1-\alpha }{2}.$$

## 4. Comparison with α-Divergences on Discrete Probability Distributions

In this section, for α≠±1, let $D^{\left(\alpha \right)}$ be an α-divergence on the positive orthant $A_{+}^{n+1}$ (which forms a convex cone) defined by

(17) $$D^{\left(\alpha \right)}\left(a,b\right)=\frac{4}{1-\alpha^{2}}\left(\frac{1-\alpha }{2}\sum_{i=1}^{n+1}a_{i}+\frac{1+\alpha }{2}\sum_{i=1}^{n+1}b_{i}-\sum_{i=1}^{n+1}a_{i}^{\frac{1-\alpha }{2}}b_{i}^{\frac{1+\alpha }{2}}\right),\;\;a,b\in A_{+}^{n+1}.$$

The α-divergence of Proposition 1 (ii) coincides with Equation (17) in the one-dimensional case.

Let S be the *n*-dimensional probability simplex, i.e.,

$$S=\left\{a=\left(a_{1},\ldots ,a_{n+1}\right)\;|\;a_{i}>0,a_{i}\in A,i=1,\ldots ,n+1,\sum_{i=1}^{n+1}a_{i}=1\right\},$$

where $a_{1},\cdots ,a_{n+1}$ represent the probabilities of n+1 states. Then, $A_{+}^{n+1}$ can be identified with the extended parameter space based on S as

(18) $$A_{+}^{n+1}=\underset{u>0}{⋃}\;\left\{a=\left(a_{1},\ldots ,a_{n+1}\right)\;|\;a_{i}>0,a_{i}\in A,i=1,\ldots ,n+1,\sum_{i=1}^{n+1}a_{i}=u\right\}.$$

Equation (18) describes the foliation of unnormalized probability simplexes. Consequently, the following corollary holds in a similar manner to Theorem 1.

**Corollary 1.** 
*For α≠±1, the α-divergence $D^{\left(\alpha \right)}$ on $A_{+}^{n+1}$ satisfies*

(19) $$D^{\left(\alpha \right)}\left(a,c\right)=\mu \;D^{\left(\alpha \right)}\left(a,b\right)+D^{\left(\alpha \right)}\left(b,c\right),\;\;\;\;\mu ={\left(\frac{u_{c}}{u_{b}}\right)}^{\frac{1+\alpha }{2}},\;\;\;\;a,\;b,and\;c\in A_{+}^{n+1},$$

*if $u_{a}=u_{b}$ and $\left(1/u_{b}\right)\;b=\left(1/u_{c}\right)\;c$, where*

$$\sum_{i=1}^{n+1}a_{i}=u_{a},\;\;\;\;\sum_{i=1}^{n+1}b_{i}=u_{b},\;\;\;\;\sum_{i=1}^{n+1}c_{i}=u_{c}.$$

For q=(1−α)/2, the divergence $D^{\left(\alpha \right)}$ restricted to the probability simplex S satisfies

$$D^{\left(\alpha \right)}\left(a,b\right)=\frac{1}{q}K_{q}\left(a,b\right),\;\;\;\;a,b\in S,$$

where $K_{q}$ is the Tsallis relative entropy defined by

$$K_{q}\left(a,b\right)\equiv -\sum_{i=1}^{n+1}a_{i}\ln_{q}\left(\frac{b_{i}}{a_{i}}\right)=\frac{1}{1-q}\left(1-\sum_{i=1}^{n+1}a_{i}^{q}b_{i}^{1-q}\right),\;a,b\in S.$$

As in the case of continuous distributions, the Tsallis relative entropy $K_{q}$ converges to the Kullback–Leibler divergence as q→1. From an information-geometric perspective, the α-divergence $D^{\left(\alpha \right)}$ also converges to the Kullback–Leibler divergence as α→−1. On the probability simplex S, the dualistic structure is induced by the α-divergence (or equivalently, the Tsallis relative entropy) [1,2,9,12,13].

## 5. Escort Distributions via Affine Immersions Generated by Exponential Families

In this section, we discuss *q*-escort distributions formed by an exponential family. Although our explanation is based on Section 2 and Section 3, there are cases in which *q*-escort distributions are mapped into the extended parameter space Θ instead of ordinary exponential distributions.

For $p\left(x;\theta_{\left(1\right)}\right)\in S_{1}$ and 0<q<1, the *q*-escort distribution $P_{q}\left(x\right)$ is defined by

$$P_{q}\left(x\right)\equiv \frac{p{\left(x;\theta_{\left(1\right)}\right)}^{q}}{\int p{\left(x;\theta_{\left(1\right)}\right)}^{q}dx}=\frac{\frac{1}{r}p{\left(x;\theta_{\left(1\right)}\right)}^{q}}{\int \frac{1}{r}p{\left(x;\theta_{\left(1\right)}\right)}^{q}dx},\;\;x\in X,$$

where r>0 [1,2]. Let $\psi_{q}$ be a function on the extended parameter space Θ defined by

$$\psi_{q}\left(a\right)=\frac{1}{1-q}\int {\left(qu_{a}\;p\left(x;\frac{1}{u_{a}}\theta_{\left(u_{a}\right)}\right)\right)}^{\frac{1}{q}}\;dx,\;\;a=\left(\theta_{a},u_{a}\right)\in \Theta ,\;\theta_{a}\in \Theta_{\left(u_{a}\right)},\;u_{a}\in A_{+}.$$

Then, the image $f_{q}\left(S_{1}\right)$ is a level surface of $\psi_{q}$ satisfying $\psi_{q}\left(a\right)=1/\left(1-q\right)$, where the affine immersion $f_{q}$ of $S_{1}$ into $A^{n}⊗A_{+}$ is defined by

(20) $$f_{q}:p\left(x;\theta_{\left(1\right)}\right)\mapsto \left(\theta_{\left(u\right)},u\right)\in \Theta_{\left(u\right)}⊗\left\{u\right\}\subset \Theta \subset A^{n}⊗A_{+},\;\;u=\int \frac{1}{q}p{\left(x;\theta_{\left(1\right)}\right)}^{q}\;dx,$$

and $\left(\theta_{\left(u\right)},u\right)$ is the *u* times of the α-coordinate of $\exists \left({\tilde{\theta }}_{\left(1\right)},1\right)\in S_{1}$, q=(1−α)/2.

In fact, for $\left(\theta_{\left(u\right)},u\right)\in f_{q}\left(S_{1}\right)$, it holds that

$$\begin{array}{ccc} \theta_{\left(u\right)}^{i} & = & \int \frac{1}{q}p{\left(x;\theta_{\left(1\right)}\right)}^{q}\left(c_{i}\left(x\right)-\frac{\partial \Phi (\theta_{\left(1\right)})}{\partial \theta_{\left(1\right)}^{i}}\right)\;dx\\ & = & u\int p{\left(x;{\tilde{\theta }}_{\left(1\right)}\right)}^{\frac{1-\alpha }{2}}\left(c_{i}\left(x\right)-\frac{\partial \Phi (\theta_{\left(1\right)})}{\partial \theta_{\left(1\right)}^{i}}\right)\;dx,\;\;\;\;i=1,\cdots ,n. \end{array}$$

As mentioned above, the unnormalized *q*-escort distribution family generated by an exponential family can be identified with $f_{q}\left(S_{1}\right)$. Similarly, the unnormalized (1−q)-escort distribution family is associated with $f_{1-q}\left(S_{1}\right)$. The image $f_{1-q}\left(S_{1}\right)$ is a level surface of $\psi_{1-q}$ satisfying $\psi_{1-q}\left(a\right)=1/q$, where $f_{1-q}$ is defined by Equation (20) with *q* replaced by 1−q. Since 1−q=(1+α)/2, the pullback of $f_{1-q}\left(S_{1}\right)$ can be regarded as the (−α)-coordinate system of the exponential family $S_{1}$.

For 0<q<1, if the Hessian matrix of the function $\psi_{q}$ is non-degenerate, $\psi_{q}$ induces the Hessian structure $\left(\Theta ,D,h\equiv \left(\partial^{2}\psi_{q}/\partial \theta^{i}\partial \theta^{j}\right)\right)$, where *D* is the canonical flat affine connection, i.e., $Dd\theta^{i}=0$ [9,18], $(\theta^{1},\cdots ,\theta^{n+1})\in \Theta$, and $\theta^{n+1}=u\in A_{+}$. By definition,

$$h\left(\frac{\partial }{\partial \theta^{i}},\frac{\partial }{\partial \theta^{j}}\right)=\frac{\partial^{2}\psi_{q}}{\partial \theta^{i}\partial \theta^{j}},\;\;\;\;\;h\left(D_{\frac{\partial }{\partial \theta^{i}}}^{\left(\alpha \right)}\frac{\partial }{\partial \theta^{j}},\frac{\partial }{\partial \theta^{k}}\right)=\frac{\partial^{3}\psi_{q}}{\partial \theta^{i}\partial \theta^{j}\partial \theta^{k}},\;\;\;\;\;\;i,j,k=1,\cdots ,n+1,$$

the tetrad $\left(\Theta ,D,D^{\left(-1\right)},h\right)$ forms a dually flat structure. The connection $D^{\left(0\right)}$ coincides with the Levi–Civita connection of the Riemannian metric *h*.

The dual coordinate system of $\left(\theta_{1},\cdots ,\theta_{n+1}\right)$ is induced by partial differentials of the following functional:

(21) $$\frac{\partial \psi_{q}}{\partial z}\left\{\frac{1}{1-q}\int {\left(qz\right)}^{\frac{1}{q}}\;dx\right\}{|}_{z=up(x;\frac{1}{u}\theta_{\left(u\right)})}=\frac{1}{1-q}\int {\left(q\;u\;p\left(x;\frac{1}{u}\theta_{\left(u\right)}\right)\right)}^{\frac{1-q}{q}}\;dx.$$

A function $z=up(x;\left(1/u\right)\theta_{\left(u\right)})$ can be replaced with ${\left(\left(1/q\right)\;p\left(x;\theta_{\left(1\right)}\right)\right)}^{q}$ on a level surface $\left(f_{q}\left(S_{1}\right),D,h\right)$. Then, the right-hand side of Equation (21) becomes

$$\frac{1}{1-q}\int p\left(x;\theta_{\left(1\right)}\right){)}^{1-q}\;dx,\;a=\left(\theta_{a},u_{a}\right)\in f_{q}\left(S_{1}\right).$$

Therefore, on a level surface $\left(f_{q}\left(S_{1}\right),D,h\right)$, the dual coordinate system of the Hessian structure $\left(\Theta ,D,h\equiv \left(\partial^{2}\psi_{q}/\partial \theta^{i}\partial \theta^{j}\right)\right)$ coincides with the (−α)-coordinate system defined by an affine immersion $f_{1-q}$.

Generally, the submanifold structure of $f_{q}\left(S_{1}\right)$ induced by $\left(\Theta ,D,D^{\left(-1\right)},h\right)$ coincides with the dualistic structure induced by the equiaffine immersion $\left(f_{q},E_{q}\right)$, where $E_{q}\equiv -d\psi_{q}{\left(\tilde{E}\right)}^{-1}\tilde{E}$ for the gradient vector field $\tilde{E}$ of $\psi_{q}$ on Θ, and $h\left(\tilde{X},\tilde{E}\right)=d\psi_{q}\left(\tilde{X}\right)$ for $\tilde{X}\in X\left(\Theta \right)$ [19,20,21,22,23].

Furthermore, $\left(f_{q}\left(S_{1}\right),D,h\right)$ has a constant curvature $\kappa =q\left(1-q\right)=\left(1-\alpha^{2}\right)/4$ [13].

## 6. Extended Divergences on Foliations by Escort Distributions

In this section, we describe a foliation of escort distributions on an extended parameter space and define an extended divergence on this foliation.

Let $F={⋃}_{0<q<1}f_{q}\left(S_{1}\right)\subset A^{n}⊗A_{+}$, where each leaf $f_{q}\left(S_{1}\right)$ is considered as a *q*-escort distribution family generated by an exponential family.

**Definition 2.** 
*Let $\psi_{q}$ be a function on the extended parameter space* Θ *defined by*

$$\psi_{q}\left(a\right)=\frac{1}{1-q}\int {\left(qu_{a}\;p\left(x;\frac{1}{u_{a}}\theta_{\left(u_{a}\right)}\right)\right)}^{\frac{1}{q}}\;dx,\;\;a=\left(\theta_{a},u_{a}\right)\in \Theta ,\;\theta_{a}\in \Theta_{\left(u_{a}\right)},\;u_{a}\in A_{+},$$

*and $f_{q}\left(S_{1}\right)$ the image of a level surface of $\psi_{q}$ satisfying $\psi_{q}\left(a\right)=1/\left(1-q\right)$, where the affine immersion of $S_{1}$ into $A^{n}⊗A_{+}$ is defined by*

$$f_{q}:p\left(x;\theta_{\left(1\right)}\right)\mapsto \left(\theta_{\left(u\right)},u\right)\in \Theta_{\left(u\right)}⊗\left\{u\right\}\subset \Theta \subset A^{n}⊗A_{+},\;\;u=\int \frac{1}{q}p{\left(x;\theta_{\left(1\right)}\right)}^{q}\;dx,$$

*and $\left(\theta_{\left(u\right)},u\right)$ is the u times of the α-coordinate of $\exists \left({\tilde{\theta }}_{\left(1\right)},1\right)\in S_{1}$, q=(1−α)/2. Then, an extended divergence $\rho_{fol}$ on the foliation F is defined as a function on F×F given by*

$$\rho_{fol}\left(a,b\right)\equiv \psi_{q_{a}}\left(a\right)-\psi_{q_{b}}\left(b\right)-\int \frac{1}{1-q_{b}}{\left(u_{b}\;p\left(x;\frac{1}{u_{b}}\theta_{b}\right)\right)}^{1-q_{b}}$$

$$\left\{\frac{1}{q_{a}}{\left(u_{a}\;p\left(x;\frac{1}{u_{a}}\theta_{a}\right)\right)}^{q_{a}}-\frac{1}{q_{b}}{\left(u_{b}\;p\left(x;\frac{1}{u_{b}}\theta_{b}\right)\right)}^{q_{b}}\right\}\;dx$$

$$\begin{array}{c} for\;\;a\in f_{q_{a}}\left(S_{1}\right),\;\;b\in f_{q_{b}}\left(S_{1}\right),\;\;0<q_{a}<1,\;\;\;0<q_{b}<1. \end{array}$$

On the level surface $\left(f_{q}\left(S_{1}\right),D,h\right)$, the restricted divergence from the canonical divergence of $\left(A_{+}^{n+1},D,h\right)$ coincides with the geometric divergence for the affine immersion $\left(f_{q},E_{q}\right)$ [19,20]. The pullback divergence to $S_{1}$ coincides with $D^{\left(\alpha \right)}$.

Let $\rho_{q}$ be the divergence on $f_{q}\left(S_{1}\right)$ defined by the affine immersion $\left(f_{q},E_{q}\right)$ in Section 5. We identify the dual space $A_{n+1}^{*}$ with $A^{n+1}$. For each *q*, the dual coordinates are defined by

(22) $$\eta_{i}\left(b\right)\equiv \frac{\partial \psi_{q_{b}}\left(b\right)}{\partial \theta^{i}},\;\;i=1,\cdots ,n+1,\;\;\;\;\left(\theta^{1},\cdots ,\theta^{n+1}\right)\in \Theta ,\;\;\theta^{n+1}=u\in A_{+}.$$

By direct calculation, we obtain the following results.

**Proposition 3.** 
*For $0<q_{b}<1$, the dual coordinates of a level surface $\left(f_{q}\left(S_{1}\right),D,h\right)$ are given by*

(23) $$\eta_{i}=\frac{1}{(1-q)q}\cdot \frac{1}{u}\int {\left(qu\;p\left(x;\frac{1}{u}\theta \right)\right)}^{\frac{1}{q}}\left(c_{i}\left(x\right)-\frac{\partial \Phi (\theta_{\left(1\right)})}{\partial \theta_{\left(1\right)}^{i}}{|}_{\theta_{\left(1\right)}=\frac{1}{u}\theta }\right)\;dx,\;\;i=1,\cdots ,n,$$

(24) $$\eta_{n+1}=\frac{1}{(1-q)q}\cdot \frac{1}{u}\int {\left(qu\;p\left(x;\frac{1}{u}\theta \right)\right)}^{\frac{1}{q}}\left\{1-\frac{1}{u}\sum_{i=1}^{n}\theta^{i}\left(c_{i}\left(x\right)-\frac{\partial \Phi (\theta_{\left(1\right)})}{\partial \theta_{\left(1\right)}^{n+1}}{|}_{\theta_{\left(1\right)}=\frac{1}{u}\theta }\right)\right\}\;dx.$$

**Proof.**  Using the techniques described in Section 3, Equation (23) is derived. By Equation (14), it holds that

$$\begin{array}{ccc} \eta_{n+1} & = & \frac{\partial \psi_{q_{b}}\left(b\right)}{\partial \bar{u}}-\frac{1}{u}\sum_{i=1}^{n}\theta^{i}\frac{\partial \psi_{q_{b}}\left(b\right)}{\partial \theta^{i}}\\ & = & \frac{1}{(1-q)q}\cdot \frac{1}{u}\int {\left(qu\;p\left(x;\frac{1}{u}\theta \right)\right)}^{\frac{1}{q}}\;dx-\frac{1}{u}\sum_{i=1}^{n}\theta^{i}\eta_{i}. \end{array}$$

Substituting Equation (23) into this relation yields Equation (24). □

By changing from the integral notation to the coordinate component notation, the following properties hold.

**Proposition 4.** 
*The extended divergence $\rho_{fol}$ on F satisfies the following properties:*

*(i)*  *If $a,b\in f_{q_{a}}\left(S_{1}\right)$, then*
$$\rho_{fol}\left(a,b\right)=\rho_{q_{a}}\left(a,b\right)=D^{\left(\alpha_{a}\right)}\left(f_{q_{a}}^{-1}\left(a\right),f_{q_{a}}^{-1}\left(b\right)\right),$$
*where $\rho_{q_{a}}$ is the divergence on $f_{q_{a}}\left(S_{1}\right)$ defined by an affine immersion, $D^{\left(\alpha_{a}\right)}$ is an $\alpha_{a}$-divergence defined by Equation (3), and $\alpha_{a}=1-2q_{a}$.*
*(ii)*  *If $q_{a}\geq q_{b}$, then*
$$\begin{array}{c} \rho_{fol}\left(a,b\right)\geq 0\;\;\;\;for\;\;\left(a,b\right)\in F\times F, \end{array}$$
*where equality holds if and only if a=b.*

**Proof.**  If $a,b\in f_{q_{a}}\left(S_{1}\right)$, then $\psi_{q_{a}}\left(a\right)=\psi_{q_{b}}\left(a\right)=\psi_{q_{b}}\left(b\right)$. By Definition 2 and the properties of affine immersions, it follows that

$$\rho_{fol}\left(a,b\right)=-\sum_{i=1}^{n+1}\eta_{i}\left(b\right)\left(\theta^{i}\left(a\right)-\theta^{i}\left(b\right)\right)=\rho_{q_{a}}\left(a,b\right),$$

which proves (i).

Next, if $1>q_{a}\geq q_{b}>0$, then we have $\psi_{q_{a}}\left(a\right)\geq \psi_{q_{b}}\left(b\right)$ from

(25) $$\psi_{q_{a}}\left(a\right)=\frac{1}{1-q_{a}},\;\psi_{q_{b}}\left(b\right)=\frac{1}{1-q_{b}}$$

by the definition of $f_{q}\left(S_{1}\right)$. Geometrically, $f_{q_{a}}\left(S_{1}\right)$ and $f_{q_{b}}\left(S_{1}\right)$ are convex surfaces centered at the origin of $A_{+}^{n+1}$, and the surface $f_{q_{a}}\left(S_{1}\right)$ lies closer to the origin than $f_{q_{b}}\left(S_{1}\right)$. Therefore, we obtain $-\sum_{i=1}^{n+1}\eta_{i}\left(b\right)\left(\theta^{i}\left(a\right)-\theta^{i}\left(b\right)\right)\geq 0$, which proves (ii). □

The extended dual divergence $\rho_{\mathrm{fol}}^{*}$ of $\rho_{\mathrm{fol}}$ is defined in the same manner as that for discrete escort distributions [15,16].

The proposed extended divergence is closely related to the duo Bregman (pseudo)divergence, where the parameters also define the underlying convex functions [24,25].

## 7. Decomposition of an Extended Divergence

In this section, we propose a decomposition theorem of an extended divergence. The following discussion also characterizes the extended divergence associated with *q*-escort distribution families under Definition 3.

We consider the flow on $F={⋃}_{0<q<1}\;f_{q}\left(S_{1}\right)$ defined by

(26) $$\frac{d\eta_{i}}{dt}=\eta_{i},\;\;\;\;i=1,\cdots ,n+1,$$

where each function $\eta_{i}$ on F represents the *i*-th component of the dual coordinate on $f_{q}\left(S_{1}\right)$ for each 0<q<1. On a level surface, because the dual coordinate is parallel to the gradient of $f_{q}$, $\left(\eta_{1},\ldots ,\eta_{n+1}\right)$ is orthogonal to $f_{q}\left(S_{1}\right)$. Then, an integral curve of Equation (26) is orthogonal to $f_{q}\left(S_{1}\right)$ for each *q* with respect to the pairing 〈·,·〉 on $A^{n+1}$ and $A_{n+1}^{*}$. The set of integral curves becomes the orthogonal foliation $F^{⊥}$ of F.

Translating into the primal coordinate system yields the following equations on F.

(27) $$\frac{d\theta^{i}}{dt}={\tilde{E}}^{i},\;\;\;\;i=1,\cdots ,n+1$$

(28) $${\tilde{E}}^{i}\equiv {\tilde{E}}_{q}^{i}=\sum_{j=1}^{n+1}h_{q}^{ij}\frac{\partial \psi_{q}}{\partial \theta^{j}}\;\;\mathrm{if}\;\;\left(\theta^{i}\right)\in f_{q}\left(S_{1}\right),$$

where $\left(h_{q}^{ij}\right)$ is the inverse matrix of $\left(h_{q\;ij}\right)$. A leaf of $F^{⊥}$ is an integral curve of the vector field $\tilde{E}$ that represents the value ${\tilde{E}}_{q}$ on $f_{q}\left(S_{1}\right)$ for each *q*.

The following theorem describes the decomposition of the extended divergence.

**Theorem 2.** 
*Let $S_{1}$ be an exponential family, and let $\left(f_{q}\left(S_{1}\right),D,h_{q}=Dd\psi_{q}\right)$ be the 1-conformally flat statistical manifold generated by the affine immersion $\left(f_{q},E_{q}\right)$, where $f_{q}$ is defined by Equation (20). Let $E_{q}\equiv -d\psi {\left({\tilde{E}}_{q}\right)}^{-1}{\tilde{E}}_{q}$, ${\tilde{E}}_{q}^{i}\equiv \sum_{j=1}^{n+1}g_{q}^{ij}\;\partial \psi_{q}/\partial \theta^{j}$, where $g_{q}$ is the restriction of $\left(g_{q\;ij}\right)=Dd\psi_{q}$ to $f_{q}\left(S_{1}\right)$. For $a,b\in f_{q_{a}}\left(S_{1}\right)$ with $0<q_{a}<1$, and $c\in F\equiv {⋃}_{0<q<1}\;f_{q}\left(S_{1}\right)$. If there exists an orthogonal leaf $L^{⊥}\in F^{⊥}$ containing both b and c, then we have*

(29) $$\rho_{fol}\left(a,c\right)=\mu \;\rho_{fol}\left(a,b\right)+\rho_{fol}\left(b,c\right),\;\;\;\;\eta \left(c\right)=\mu \;\eta \left(b\right),\;\;\mu >0,$$

*where η(·) denotes the dual coordinate of $f_{q}\left(S_{1}\right)$ for each q.*

**Proof.**  Because $a,b\in f_{q_{a}}\left(S_{1}\right)$, it follows that $\psi_{q_{a}}\left(a\right)=\psi_{q_{b}}\left(b\right)$ with $q_{b}=q_{a}$. By Definition 2, we obtain

$$\begin{array}{ccc} \rho_{fol}\left(a,c\right) & = & \psi_{q_{a}}\left(a\right)-\psi_{q_{c}}\left(c\right)-\sum_{i=1}^{n+1}\eta_{i}\left(c\right)\left(\theta^{i}\left(a\right)-\theta^{i}\left(c\right)\right)\\ & = & \psi_{q_{b}}\left(b\right)-\psi_{q_{c}}\left(c\right)-\sum_{i=1}^{n+1}\left\{\eta_{i}\left(c\right)\left(\theta^{i}\left(a\right)-\theta^{i}\left(b\right)\right)+\eta_{i}\left(c\right)\left(\theta^{i}\left(b\right)-\theta^{i}\left(c\right)\right)\right\}\\ & = & -\mu \sum_{i=1}^{n+1}\eta_{i}\left(b\right)\left(\theta^{i}\left(a\right)-\theta^{i}\left(b\right)\right)\\ & & +\{\psi_{q_{b}}\left(b\right)-\psi_{q_{c}}\left(c\right)-\sum_{i=1}^{n+1}\eta_{i}\left(c\right)\left(\theta^{i}\left(b\right)-\theta^{i}\left(c\right)\right)\}\\ & = & \mu \;\rho_{fol}\left(a,b\right)+\rho_{fol}\left(b,c\right). \end{array}$$

□

In a manner similar to discrete escort distributions [15,16,26], we obtain the gradient flow on a leaf $f_{q}\left(S_{1}\right)$ using the extended divergence.

Finally, an example of a normal distribution is shown. A normal distribution is defined by

$$\begin{array}{c} p\left(x;\;\mu ,\sigma^{2}\right)=\frac{1}{\sqrt{2\pi }\sigma }\exp\left(-\frac{{\left(x-\mu \right)}^{2}}{2\sigma^{2}}\right). \end{array}$$

The affine coordinate system $\left(\theta^{1},\theta^{2}\right)$ is defined as

$$\begin{array}{c} p\left(x;\;\theta^{1},\theta^{2}\right)=\exp\left(\theta^{1}x+\theta^{2}x^{2}-\frac{{\left(\theta^{1}\right)}^{2}}{4\theta^{2}}-\log\sqrt{2\pi }\sigma \right), \end{array}$$

where

$$\begin{array}{c} \theta^{1}=\frac{\mu }{\sigma^{2}},\;\;\;\;\theta^{2}=-\frac{1}{2\sigma^{2}}. \end{array}$$

**Figure 3:**
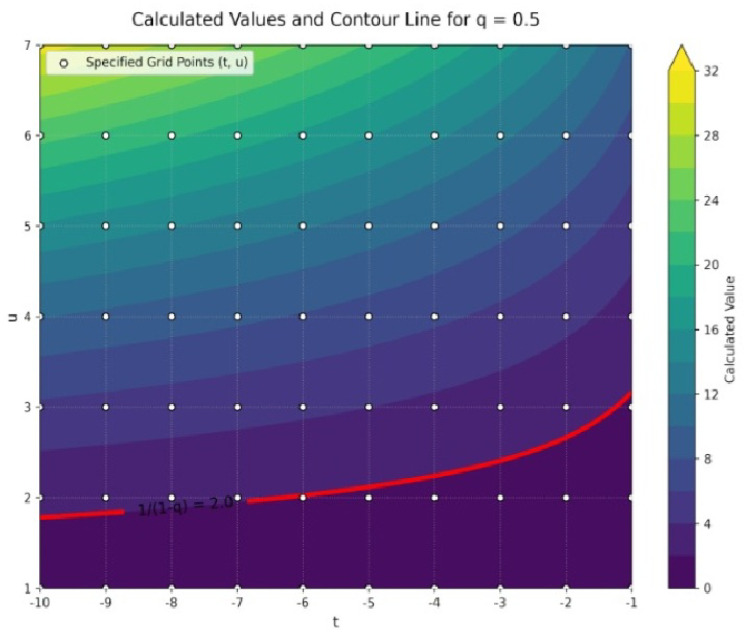
Contour lines of a potential function $\psi_{q}$ (q=0.5). The thick line is for 1/(1−q)=2 (horizontal axis: $\theta^{2}$, vertical axis: *u*).

**Figure 4:**
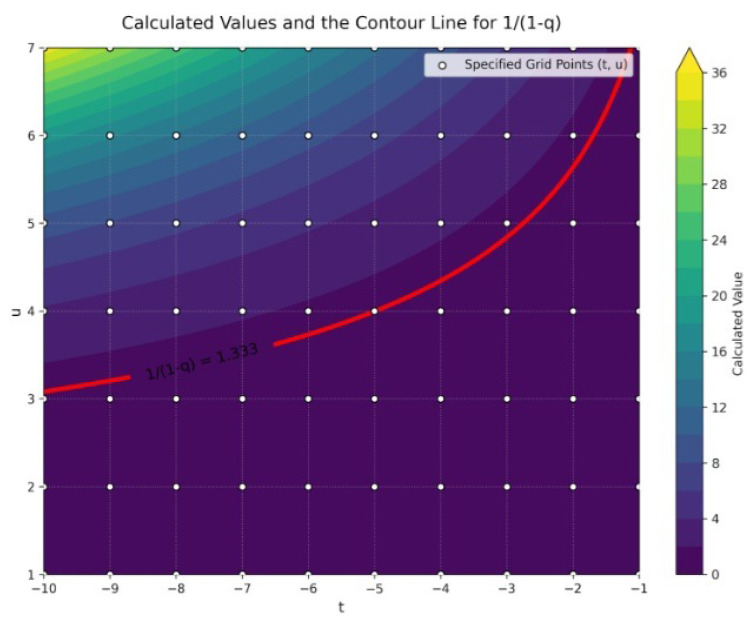
Contour lines of a potential function $\psi_{q}$ (q=0.25). The thick line is for 1/(1−q)=1.333. (horizontal axis: $\theta^{2}$, vertical axis: *u*).

**Figure 5:**
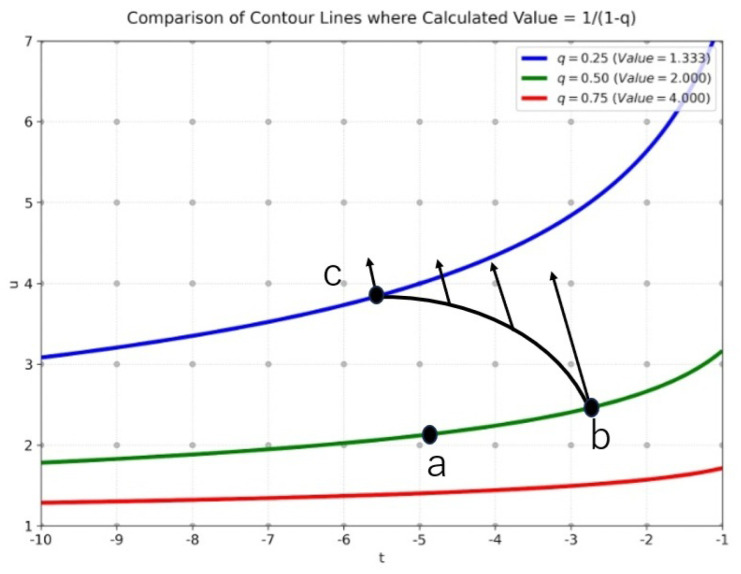
Level surfaces that realize the *q*-escort distribution family (from the bottom in order, in the case of q=0.75,0.5, and 0.25) and a gradient flow from *c* to *b*. The arrows are in the direction of the gradient of level surfaces and represent the dual coordinates at each point. The case of u=1 shows the exponential family (q=1). (horizontal axis: $\theta^{2}$, vertical axis: *u*).

**Figure 6:**
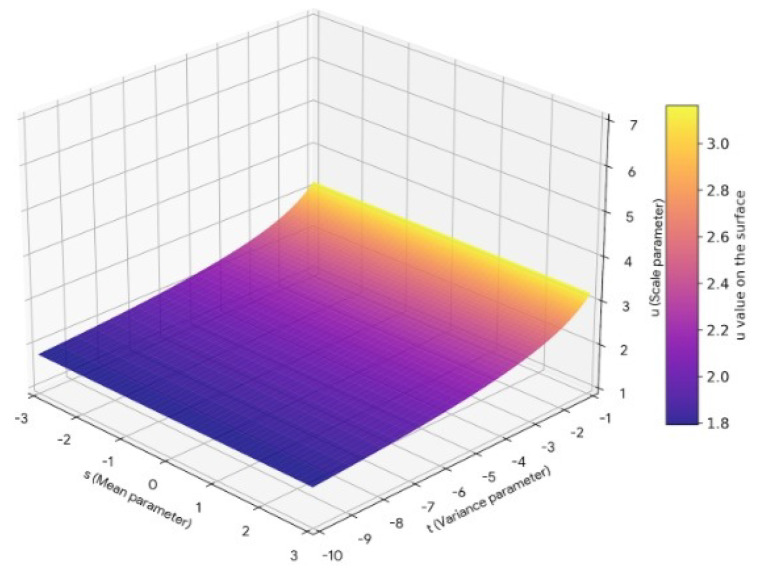
A level surface that realizes the *q*-escort distribution family for q=0.5. (horizontal axis: $\theta^{1}$, $\theta^{2}$, vertical axis: *u*).

When $\theta^{1}=0$, the values of $\psi_{q}$ and level surfaces for $\psi_{q}=1/\left(1-q\right)$ are shown in Figure 3 (q=0.5) and Figure 4 (q=0.25). The level surfaces overlaid for *q* = 0.75, 0.5, and 0.25 is shown in Figure 5. Figure 6 shows the level surface for q=0.5, taking $\theta^{1}$ into consideration. It is impossible to create a gradient flow in the directions where the Hessian of $\psi_{q}$ does not degenerate. In directions where it degenerates, it is necessary to define a separate dual geometric structure.

## 8. Discussion

This study investigated a foliation of deformed exponential families corresponding to sets of escort distributions with *q*-parameters, accommodating the continuous transition of α-parameters within the framework of information geometry. By establishing a natural definition for this foliation, we provide an account of an extended divergence that quantifies the proximity between *q*-escort distributions characterized by different *q*-parameters.

In the future, the cases of 1≤q<3, which is important in nonequilibrium statistical mechanics, and ∞<q≤0 must be considered. Accurately comparing the *q*-escort and *q*-exponential families also remains a challenge.

This theoretical framework offers a robust mathematical foundation for advancing machine learning and deep learning methodologies, particularly in systems where heterogeneous *q*-parameters coexist. In the context of deep learning, our results are directly applicable to the design of robust loss functions and *q*-generalized activation functions (such as the *q*-softmax), which are essential for handling heavy-tailed noise and non-Gaussian data distributions. Furthermore, the proposed decomposition theorem provides an information-geometric basis for adaptive optimization algorithms, potentially allowing the dynamic tuning of *q*-parameters during the training process to enhance model generalization and convergence. While the decomposition theorem suggests a structural path toward determining optimal *q*-parameters, the specific algorithmic implementations for hyperparameter optimization warrant further investigation. Exploring the connection between our framework and the recently proposed λ-duality in nonextensive statistical mechanics [27,28] remains a promising direction for future research in both statistical physics and neural network theory.

## Data Availability

No new data were created or analyzed in this study. Data sharing is not applicable to this article.
